# Supplementary material for: Systemic Steroids in Preventing Bronchopulmonary Dysplasia (BPD): Neurodevelopmental Outcome According to the Risk of BPD in the EPICE Cohort
Source: Int J Environ Res Public Health. 2022 May 5;19(9):5600. doi: 10.3390/ijerph19095600 (PMC9106050; doi:10.3390/ijerph19095600)
Supplement: Supplementary file 1 [file ijerph-19-05600-s001.zip › ijerph-1639502-supplementary.pdf]

Supplementary Materials:

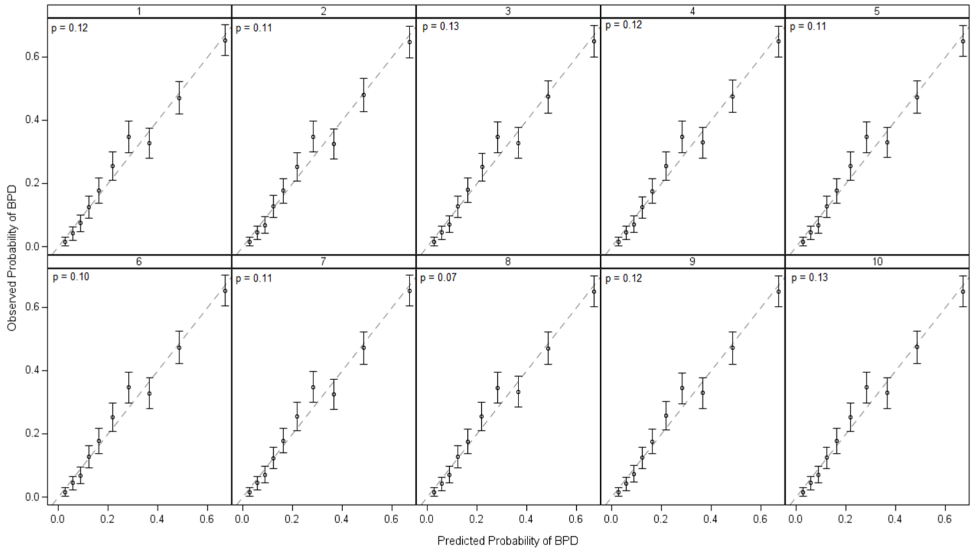

Supplemental Figure S1: Calibration plot of the prediction model for BPD in each imputation dataset.

Supplemental Table S1: Characteristics of neonates included in the study and infants who died before day 14.

Variables	Infants alive at day 14 (n=3662)	Infants who died before day 14 (n=434)	p-value
**Neonatal characteristics**			
Gestational age (weeks) [1]	28 (26 to 29)	26 (24 to 27)	**<0.001**
Boys	1982 / 3662 (54.1)	247 / 434 (56.9)	0.27
Birth weight (g) [2]	1000 (810 to 1200)	777.5 (640 to 990)	**<0.001**
Multiple pregnancy	1075 / 3662 (29.4)	137 / 434 (31.6)	0.07
SGA	395 / 3662 (10.8)	37 / 434 (8.5)	0.07
Inborn	3240 / 3655 (88.6)	373 / 433 (86.1)	0.12
Apgar < 7 at 5 min	1233 / 3414 (36.1)	233 / 380 (61.3)	**<0.001**
Pre-eclampsia / Eclampsia / HELLP	530 / 3592 (14.8)	52 / 424 (12.3)	0.19
PROM	915 / 3579 (25.6)	106 / 424 (25.0)	0.80
**Neonatal care**			
Antenatal steroids	3269 / 3629 (90.1)	343 / 427 (80.3)	**<0.001**
Level 3 unit	3025 / 3647 (82.9)	354 / 430 (82.3)	0.75
Surfactant	2685 / 3595 (74.7)	383 / 417 (91.8)	**<0.001**
Invasive respiratory support	2230/3628 (61.5)	351/424 (82.8)	**<0.001**
PDA requiring treatment	1046 / 3439 (30.4)	77 / 400 (19.3)	**<0.001**
IVH grade III or IV	273 / 3598 (7.6)	139 / 389 (35.7)	**<0.001**
Early onset neonatal infection	279 / 3166 (8.8)	51 / 360 (14.2)	**<0.001**
Necrotizing enterocolitis	244 / 3067 (7.9)	1 / 346 (0.3)	**<0.001**
Postnatal steroids (respiratory indication)	54 / 3662 (1.5)	1 / 434 (0.2)	**<0.001**

Values are presented as n / N (percentage) or median (interquartile range).

1 available for 4 096 patients

2 available for 3 825 patients

Abbreviations: SGA, small for gestational age; HELLP, Hemolysis Elevated Liver Enzymes Low Platelet count; PROM, prolonged rupture of membranes; MV, mechanical ventilation; CPAP, continuous positive airway pressure; PDA, patent ductus arteriosus; IVH, Intraventricular Hemorrhage.

Supplemental Table S2: PNS use for infants included in the EPICE cohort.

	**Dexamethasone 207 (37.1%)**	**Hydrocortisone 182 (32.6%)**	**Betamethasone 81(14.5%)**
Initial dose (mg/kg/d)	0.2 (0.15–0.5)	4.0 (1.3–5)	0.2 (0.1–0.3)
Age at initiation (d)	24 (15–38)	12 (8–20)	21 (17–30)
Treatment duration (d)	11 (6–22)	21 (12–33)	6 (3–12)
Mean GA at birth (sd)	25.6 (1.4)	26.2 (1.6)	26.5 (1.6)

Data are median (Q1-Q3) unless indicated.

Supplemental Table S3: Characteristics of neonates in the group treated with PNS and the untreated group after multiple imputation.

**Variables**	**PNS (n=558)**	**Untreated (n=3104)**	**ASD (%)**
**Neonatal characteristics**			
Gestational age ≤ 28 weeks	506 (90.7)	2153 (69.44)	61.64
Boys	334 (59.8)	1648 (53.1)	16.39
Multiple pregnancy	150 (26.9)	925 (29.8)	5.15
SGA	129 (23.1)	580 (18.7)	12.15
Inborn	479 (85.8)	2766 (89.1)	4.72
Apgar < 7 at 5 min	273 (48.9)	1074 (34.6)	31.46
Pre-eclampsia / Eclampsia / HELLP	90 (16.1)	450 (14.5)	5.32
PROM	133 (23.8)	812 (26.2)	8.60
**Neonatal care**			
Antenatal steroids	485 (86.9)	2813 (90.6)	6.09
Level 3 unit	480 (86.0)	2553 (82.2)	15.48
Surfactant	509 (91.2)	2222 (71.6)	58.61
Heavy Initial respiratory support	503 (90.1)	1745 (56.2)	88.77
PDA requiring treatment	32 (5.7)	78 (2.5)	16.42
IVH grade III or IV	68 (12.2)	213 (6.9)	18.83
Early onset neonatal infection	80 (14.3)	389 (12.5)	5.89
Late onset neonatal infection	362 (64.9)	1192 (38.4)	57.98
Necrotizing enterocolitis	28 (5.0)	119 (3.8)	6.30
Postnatal steroids (respiratory indication)	54 / 3662 (1.5)	1 / 434 (0.2)	**<0.001**

Values are presented as n / N (percentage) or mean ± standard deviation.

Descriptive values and ASD were calculated after handling missing data by multiple imputation.

Abbreviations: ASD, absolute standardized difference

Supplemental Table S4: Characteristics of lost in follow up population (PARCA-R or ASQ evaluation).

**Variables**	**Lost in Follow up (PARCA-R/ASQ) (n=1884)**	**Followed Up (PARCA-R/ASQ) (n= 1778)**
**Neonatal characteristics**		
Gestational age <28weeks	1368 (72.6)	1291 (72.6)
Boys	1018 (54.0)	964 (54.2)
SGA	351 (18.6)	358 (20.1)
Inborn	1634 (86.7)	1606 (90.3)
Apgar < 7 at 5 min	614 (36.7)	619 (36.7)
**Neonatal care**		

Antenatal steroids	1634 (86.7)	1635 (92.0)
Surfactant	1412 (74.9)	1273 (71.6)
Invasive respiratory support	1164 (61.8)	1066 (60)
IVH grade III or IV	159 (8.4)	114 (6.4)
Early onset neonatal infection	161 (8.5)	118 (6.6)
Late onset neonatal infection	855 (45.4)	668 (37.6)
BPD	463 (24.6)	447 (25.1)

Values are presented as n / N (percentage).

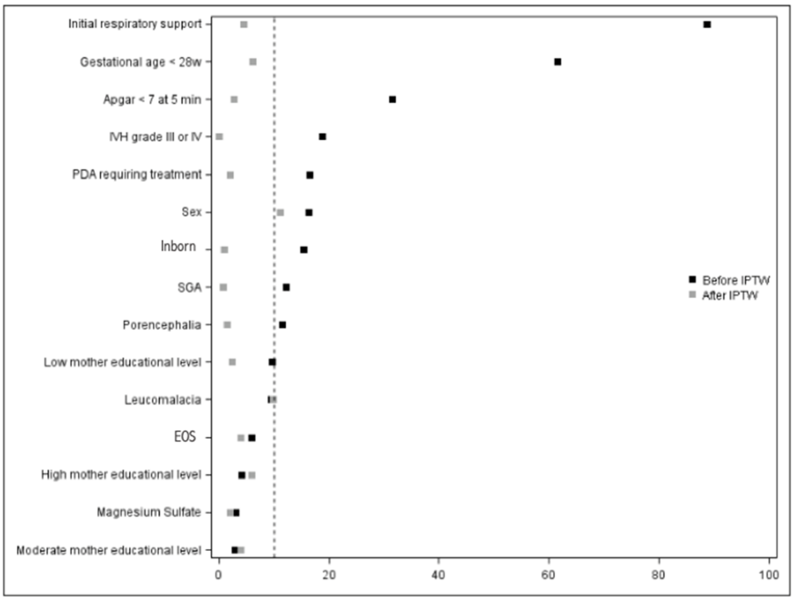

Supplemental Figure S2: Absolute standardized differences between PNS and non-PNS treated infants before and after inverse probability of treatment weighting (IPTW) adjustment after handling missing data by multiple imputation. Abbreviations: IPTW, inverse probability of treatment weighting; SGA, small for gestational age; EONI, early onset neonatal infection.

Supplemental Table S5: Characteristics of neonates treated by hydrocortisone and dexamethasone or betamethasone.

Variables	Hydrocortisone (n=171)	Dexa/Betamethasone (n=270)	p- value
**Neonatal characteristics**			
Gestational age <28weeks	17(9.9)	17(6.3)	0.18
Boys	103 (60.2)	170(63.0)	0.72
Multiple pregnancy	57(33.3)	60(22.2)	**0.03**
SGA	42(24.6)	63(23.3)	0.79
Inborn	151(88.3)	235(87.4)	0.89
Apgar < 7 at 5 min	65(39.9)	126(54.8)	0.18

Pre-eclampsia / Eclampsia / HELLP	24(14.2)	49(18.4)	0.30
PROM	33(19.5)	60(22.7)	0.51
**Neonatal care**			
Antenatal steroids	150(87.7)	242(90.0)	0.83
Surfactant	160(93.6)	252(93.3)	0.98
Invasive respiratory support	160(94.1)	248(91.9)	0.85
IVH grade III or IV	26 (15.3)	32(12.0)	0.34
Early onset neonatal infection	14(8.5)	23(9.2)	0.90
Late onset neonatal infection	101(59.1)	185(68.5)	0.23
Risk of BPD			
High	14(8.2)	20(7.5)	0.77
Moderate	42(24.7)	67(25.3)	0.95
Low	114(67.1)	178(67.2)	0.92

Values are presented as n / N (percentage).

Categorical variables were expressed as frequencies and percentages. Continuous variables were expressed as means (standard deviation) or median (interquartile range (IQR)). Normality of distribution was checked graphically and by using the Shapiro–Wilk test. To assess the potential bias related to death before day 14, the main infant's characteristics were compared between included and excluded infants using Student's t test for continuous variables and Chi-square test for categorical variables.

In order to classify at baseline the infants into three groups according to BPD risk at day 14, we firstly developed a predictive model for BPD in our study population using a multivariable mixed logistic regression model by including 15 pre-selected candidates' predictors and considering region as random effect (for the center effect). The number of death before assessment being less than 10% (158 infants, 4%), we were able to consider the outcome BPD alone. The full model was simplified using a stepwise-backward selection procedure with a removal criteria of 0.05. Before developing multivariable model, the log-linearity assumption for continuous predictors was checked using restricted cubic spline functions [30]. The performance of the predictive model was examined by assessing discrimination (by calculating the c-statistics) and calibration (by using Hosmer and Lemeshow test and calibration plot) in each imputed dataset. As our goal was to classify our population rather than publishing a new risk score, no external validation was needed. Infants were classified into three BPD risk groups by using the tertiles of probability estimated from thr predictive model. Comparison between these three BPD risk groups in anthropometric characteristics, respiratory and neurosensory outcomes at 2 years of age were made using linear mixed models for continuous variables and mixed logistic regression models for binary variables by

including the region as random effect. For linear mixed models, normality of residuals was satisfied.

As main analysis, we compared neurodevelopment outcomes at 2 years corrected age according to use of PNS in overall study population and according to BPD risk groups using mixed logistic regression models with region as random effect; odds ratios (ORs) with theirs 95% confidence intervals (CIs) were calculated as effect sizes using untreated infants as reference. Heterogeneity in PNS effect sizes according to BPD risk groups was tested by including the corresponding multiplicative interaction term into the mixed logistic regression models. To consider the potential confounding factors, comparisons in neurodevelopment outcomes were further done by using inverse probability of treatment weighting (IPTW) propensity score (PS) method (using stabilized inverse PS as weighty in mixed logistic regression models) [31]. The PS was estimated using a multivariable logistic regression model, with PNS-treatment (yes vs.no) as the dependent variable and all potential confounders selected on subject-matter knowledge using the causal directed acyclic graphs theory [32]as independent variables. The following factors were considered in PS calculation: mother's characteristics (maternal education level), newborns' characteristics (gestational age, birth weight<3th percentiles, gender), delivery' characteristics (magnesium sulfate treatment, Apgar score at 5minutes), place of birth (inborn/outborn) and neonatal hospitalization features (initial respiratory support, patent ductus arteriosus (PDA), early onset neonatal infection, high grade intra-ventricular hemorrhage, periventricular leuco-malacia (PVL), porencephalia). To evaluate the bias reduction using the IPTW method, absolute standardized differences were calculated before and after IPTW adjustment.

To avoid case deletion in multivariate regression analyses due to missing baseline and outcome data (to calculated the predictive BPD model, and to adjust the comparisons in neurodevelopment outcomes), missing data were imputed by multiple imputations using the regression-switching approach (chained equations, m=10 imputations) [33]. The imputation procedure was performed under the missing-at-random assumption using all baseline and outcome data, with the predictive mean-matching method for continuous variables and logistic regression (binary, ordinal, or multinomial) models for categorical variables. Rubin's rules were used to combine the estimates derived from multiple imputed data sets [34].

Statistical testing was conducted at the two-tailed α-level of 0.05. Data were analyzed using R (Version 3.5.1) and the SAS version 9.4 (SAS Institute, Cary, NC) software's.
